# Catecholaminergic Modulation of Metacontrol Is Reflected in Aperiodic EEG Activity and Predicted by Baseline GABA+ and Glx Concentrations

**DOI:** 10.1002/hbm.70173

**Published:** 2025-03-04

**Authors:** Yang Gao, Anna Helin Koyun, Ann‐Kathrin Stock, Annett Werner, Veit Roessner, Lorenza Colzato, Bernhard Hommel, Christian Beste

**Affiliations:** ^1^ School of Psychology Shandong Normal University Jinan China; ^2^ Cognitive Neurophysiology, Department of Child and Adolescent Psychiatry, Faculty of Medicine TU Dresden Dresden Germany; ^3^ German Center for Child and Adolescent Health (DZKJ), Partner Site Leipzig/Dresden Dresden Germany

**Keywords:** aperiodic noise, catecholamines, conflict monitoring, GABA, glutamate, metacontrol, methylphenidate

## Abstract

The ability to balance between being persistent versus flexible during cognitive control is referred to as “metacontrol” and reflected in the exponent of aperiodic neural activity. Theoretical considerations suggest that metacontrol is affected by the interplay of the GABAergic, glutamatergic, and catecholaminergic systems. Moreover, evidence suggests that fronto‐striatal structures play an important role. Yet, the nexus between neurobiochemistry and structural neuroanatomy when it comes to the foundations of metacontrol is not understood. To examine this, we investigated how an experimental manipulation of catecholaminergic signaling via methylphenidate (MHP) and baseline levels of GABA and glutamate in the anterior cingulate cortex (ACC), supplementary motor area (SMA), and striatum as assessed via MR spectroscopy altered task performance and associated aperiodic activity (assessed via EEG) during a conflict monitoring task. We investigated *N* = 101 healthy young adults. We show that the EEG‐aperiodic exponent was elevated during task performance, as well as during cognitively challenging task conditions requiring more persistent processing and was further enhanced by MPH administration. Correlation analyses also provided evidence for an important role of individual characteristics and dispositions as reflected by the observed role of GABA+ and Glx baseline levels in the ACC, the SMA, and the striatum. Our observations point to an important role of catecholamines in the amino acid neurotransmitter‐driven regulation of metacontrol and task‐specific (changes in) metacontrol biases. The results suggest an interplay of the GABA/Glx and the catecholaminergic system in prefrontal‐basal ganglia structures crucial for metacontrol.

## Graphical abstract

The study combined MR‐spectroscopy and EEG‐aperiodic activity to delineate the structural‐neurobiochemical basis of flexible adjustments in cognitive control (i.e., metacontrol).

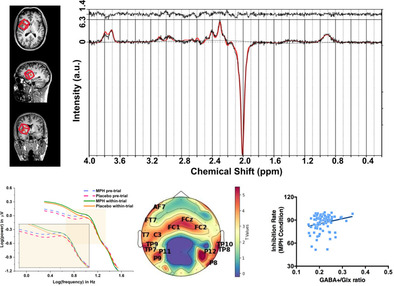

**Summary**

- Metacontrol is reflected by aperiodic neural activity
- Catecholamines shift metacontrol and the aperiodic exponent toward more stable processing. GABA+ and Glx baseline levels predict both metacontrol performance and MPH effects on it.

## 1 Introduction

Adaptive behavior often faces a control dilemma (Beste, Moll, et al. 2018; Goschke 2000; Goschke and Bolte 2014; Hommel and Colzato 2017; Hommel et al. 2024): some situations demand focused cognitive control to handle distractions, while others require flexibility in uncertain contexts. This balance, termed “metacontrol” (Hommel 2015; Hommel et al. 2024; Hommel and Colzato 2017), is central to the Metacontrol State Model (Hommel 2015). A persistence bias emphasizes goal focus, strong competition, and engagement with relevant information, whereas a flexibility bias relaxes focus and reduces competition to allow processing of irrelevant information. Evidence shows these biases adapt to task demands (Hommel and Colzato 2017; Mekern et al. 2019; Zhang et al. 2022; Pi et al. 2024; Van Schependom et al. 2024).

Recent advances in electrophysiology emphasize the critical role of aperiodic neural activity (non‐oscillatory EEG) in cognitive control, challenging its prior dismissal as mere noise (Gao et al. 2024; Groppe et al. 2013; Gyurkovics et al. 2021; Jia et al. 2024; Monchy et al. 2024; Pertermann, Bluschke, et al. 2019; Pertermann, Mückschel, et al. 2019; Wainio‐Theberge et al. 2022; Yan et al. 2024; Zhang et al. 2023). The power spectrum reveals a frequency‐dependent decline in spectral power, quantified by the aperiodic exponent—represented as the negative 1/f slope in the log‐transformed power spectrum (Donoghue et al. 2020; He 2014; Pritchard 1992). This exponent captures neural variability, reflecting the brain's capacity to adapt its responses to situational demands (Waschke, Kloosterman, et al. 2021; Waschke, Donoghue, et al. 2021). Additionally, the aperiodic exponent reflects the excitation/inhibition (*E*/*I*) ratio, where higher excitatory activity increases neural variability and reduces synchronization, while inhibitory dominance reduces variability and enhances focused processing (Gao et al. 2017; Lombardi et al. 2017; Voytek and Knight 2015). This aligns with metacontrol: a persistence bias (higher aperiodic exponent) indicates inhibitory dominance and stable processing, while a flexibility bias (lower exponent) reflects excitatory dominance and exploratory behavior (Hommel and Colzato 2017). Thus, the aperiodic exponent signals metacontrol biases, with higher values indicating persistence and lower values indicating flexibility.

In line with these considerations, the aperiodic exponent has been found to vary systematically with task conditions, increasing during persistence demands and decreasing during flexibility demands, suggesting that it reflects metacontrol biases (Gao et al. 2024; Pi et al. 2024; Yan et al. 2024; Zhang et al. 2023). Recently, we demonstrated that methylphenidate (MPH), which boosts catecholamines, increases the aperiodic exponent (Gao et al. 2024) likely by enhancing the signal‐to‐noise ratio (SNR) and reducing neural variability, thereby improving the distinction between relevant and irrelevant information (Aston‐Jones and Cohen 2005; Cohen et al. 2002; Kroener et al. 2009; Li et al. 2001; Li and Rieckmann 2014; Nieuwenhuis et al. 2005; Rolls et al. 2008; Servan‐Schreiber et al. 1990; Vander Weele et al. 2018; Winterer and Weinberger 2004; Yousif et al. 2016; Ziegler et al. 2016). Interestingly, the catecholaminergic system is closely interacting with and modulating both excitatory and inhibitory neuronal signaling (Tritsch and Sabatini 2012), as catecholamines play a role in modulating the responsiveness of GABAergic and glutamatergic synapses within the prefrontal cortex and other brain regions critical for response selection and cognitive control (Manz et al. 2021; Plenz 2003). Given the relationship between MPH‐driven catecholamine alterations and their impact on GABAergic and glutamatergic transmission, it seems likely that individual variations in the baseline levels of GABA and glutamate might predict (changes in) metacontrol as reflected by aperiodic EEG activity and its modulation by MPH.

To investigate this aspect, we reanalyzed a sample that was originally assessed and studied for other scientific purposes (Koyun et al. 2024). Following prior EEG studies (Adelhöfer et al. 2021; Zhang et al. 2023; Gao et al. 2024; Jia et al. 2024; Pi et al. 2024; Yan et al. 2024), we applied the spectral parameterization approach (Fitting Oscillations and One Over f [FOOOF]) (Donoghue et al. 2020) to analyze aperiodic exponents during MPH or placebo intake in a cognitive task combining the Simon and Go/NoGo tasks. This design involved conditions requiring varying levels of metacontrol persistence and flexibility. Since pre‐stimulus neural activity can influence task performance (Adelhöfer et al. 2021; Adelhöfer and Beste 2020; Huang et al. 2017; Northoff et al. 2024; Prochnow et al. 2022; Wainio‐Theberge et al. 2021; Wendiggensen et al. 2022; Wolff, Yao, et al. 2019; Wolff et al. 2021), we compared aperiodic exponents from the critical within‐trial period to the noncritical pretrial period. This approach separates task‐specific aperiodic activity from random fluctuations and evaluates changes in the exponent across conditions, using pretrial values as a neutral reference. We expected to replicate the findings by Gao et al. (2024) that MPH increases the aperiodic exponent in two distinct ways. First, we proposed that MPH should induce a state‐like effect, with a significant impact during both pretrial and within‐trial periods. Second, we anticipated that electrode‐specific analyses would demonstrate that MPH influences selective processes in the within‐trial period by reducing the downregulation of aperiodic activity in situations that require more metacontrol persistence. Furthermore, we hypothesized that individual differences in baseline GABAergic and glutamatergic transmission could predict changes in metacontrol in response to MPH. To test this hypothesis, we measured the baseline levels/total concentrations of GABA+ (GABA plus macromolecules) as well as Glx (glutamate plus glutamine) and their ratio as a proxy for the *E*/*I* ratio in the striatum, anterior cingulate cortex (ACC), and supplementary motor area (SMA) of healthy adults using magnetic resonance spectroscopy (MRS). Those brain areas had been selected as they are essential for metacontrol given their role in automatic motor activation, suppressing dominant action plans (ACC and SMA; [Bari and Robbins 2013] and efficient response selection ACC and striatum [Adams et al. 2017; Redgrave et al. 2011]). Accordingly, we hypothesized that GABA+ and Glx concentrations, and/or their ratio in these areas modulate the effects of MPH on metacontrol and the aperiodic exponent.

## 2 Materials and Methods

### 2.1 Participants

The current study is based on existing datasets collected by Koyun et al. (2024). Participants were identified as outliers and excluded from all analyses if they fulfilled any of the following criteria: behavioral accuracy below chance level (< 50%), technical issues during data recording, insufficient EEG signal quality after preprocessing, or missed the second appointment (see for details, Koyun et al. 2024). *N* = 101 participants (*M* = 25.26; SD = 3.03; range 20–31 years; comprising 59 males) were included in the behavioral analyses and subsequent neurophysiological and ^1^H‐MRS analyses. All participants reported no neurological or psychiatric disorders, developmental conditions affecting brain function, dairy allergies, pregnancy, and all had MRI compatibility and normal or corrected‐to‐normal vision. Participants provided written informed consent before participation and received a €60 reimbursement after completing the two study appointments. The original study was conducted in accordance with the Declaration of Helsinki and approved by the ethics commission of the Medical Faculty of TU Dresden.

### 2.2 Research Methodology

**FIGURE 1:**
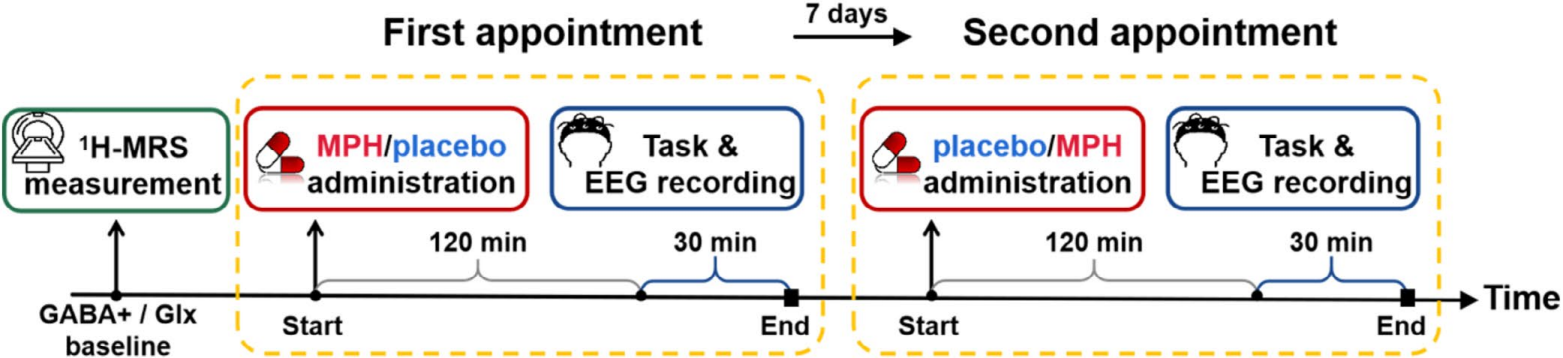
Schematic illustration of the timeline of experimental interventions for each participant. The horizontal axis represents time in minutes. Participants underwent a ^1^H‐MRS measurement before the first task appointment. The behavioral task and concurrent EEG recording started 120 min after MPH/placebo administration and lasted for approximately 30 min. Participants were administrated either MPH or a placebo at the start of each appointment, with the sequence being randomly assigned.

The study included a baseline ^1^H‐MRS measurement and two experimental EEG sessions (Figure 1). During each EEG session, participants received either the assigned MPH dose (low, medium or high dose) or a lactose placebo in a double‐blind manner. The two testing sessions were scheduled 7 days apart to prevent carryover effects and guarantee drug washout. Pseudo‐randomization ensured equal numbers of participants in each subgroup, based on pharmacological dose (low, medium, high) and the sequence of appointments (MPH on the first or second appointment). This pseudo‐randomization also achieved gender balance within and across all subgroups. Additionally, group assignments were fully randomized and double‐blind.

### 2.3 MPH Administration

On one appointment, participants were administered the assigned MPH dose (either low: 0.25 mg, medium: 0.50 mg, or high: 0.75 mg/kg body weight) and a lactose placebo on the other, in a double‐blind manner. Dosage levels were determined based on previous studies (e.g., Beste, Adelhöfer, et al. 2018) for the low (0.25 mg/kg) and medium (0.50 mg/kg) doses, and the high dose (0.75 mg/kg) set just below the recommended maximum of 0.80 mg/kg. The experiment started approximately 2 h post‐MPH/placebo administration on each appointment, aligning with the peak plasma levels at 1–3 h post‐dose, with maximum concentration around 2 h after oral ingestion (Challman and Lipsky 2000).

### 2.4 ^1^H‐MRS Data Acquisition and Processing

All MRI and MRS data were acquired using a Siemens 3T Prisma scanner (Siemens Healthineers, Erlangen, Germany) with a 32‐channel receive‐only head rf coil.

**FIGURE 2:**
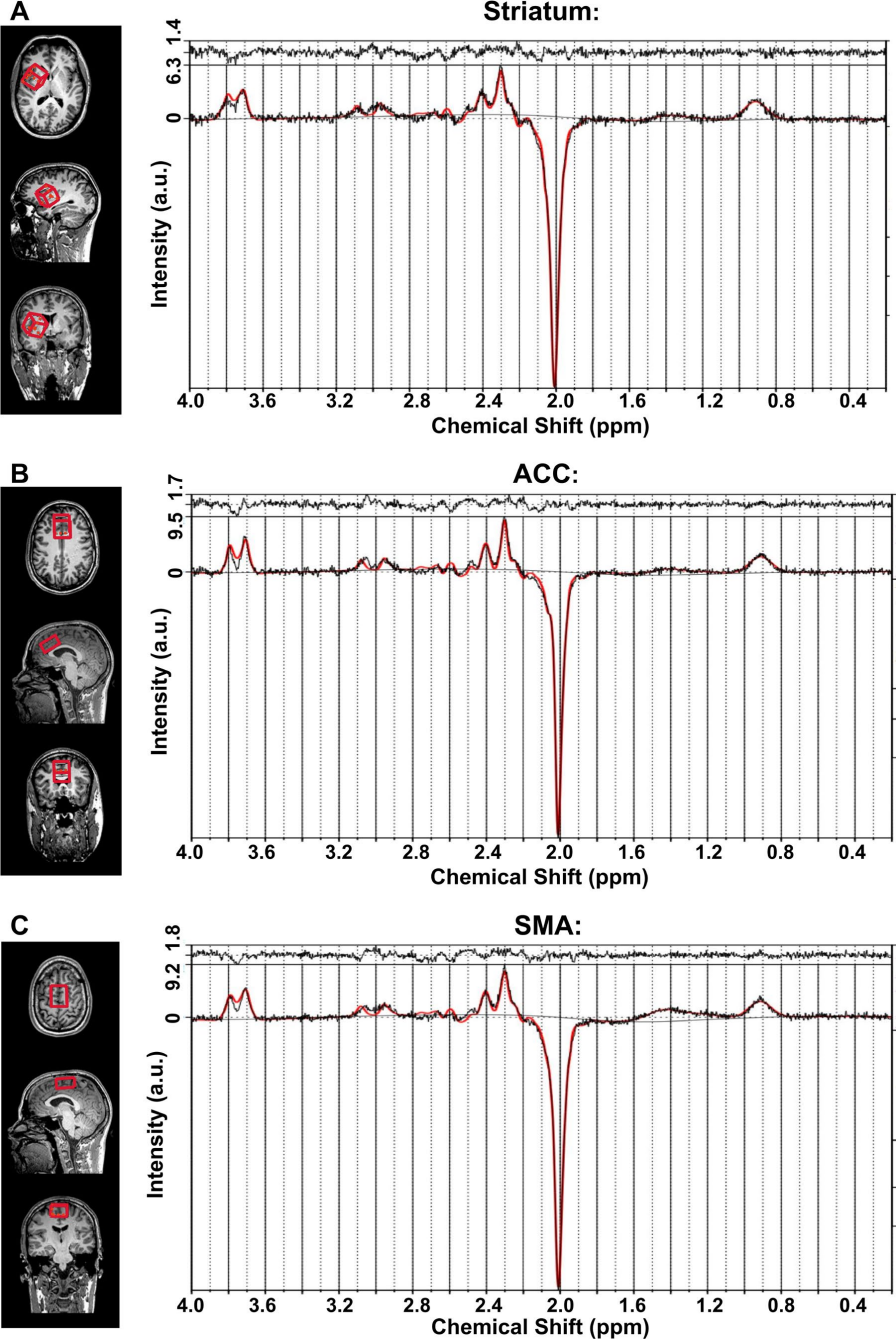
Illustration of VOI placements (left panel) and representative difference spectra (“edit on–edit off”) from MRS (right panel) in three brain regions: (A) Striatum, (B) ACC, and (C) SMA. The bottom plots show the measured spectrum (black line) overlaid with the fitted spectrum (red line). The top plot displays residuals. The *x* axis shows chemical shift (ppm) and the *y* axis represents signal intensity (arbitrary units, a.u.).

Following a localizer scan, high‐resolution 3D T1‐weighted sagittal images were acquired using the MPRAGE sequence (1 mm isovoxel) and reconstructed for precise voxel placements. A 30 × 30 × 30 mm voxel of interest (VOI) was positioned in the right striatum, a 20 × 30 × 40 mm VOI centered over the midline covered large portions of both left and right ACC (with minimal inclusion of neighboring regions), and an additional 20 × 30 × 40 mm VOI was placed to encompass both left and right (pre‐)SMA. Representative placements and spectra of all three VOIs are shown in Figure 2. ^1^H‐MRS quantified GABA+, Glx, and tCr concentrations in the striatum, ACC, and SMA, with separate VOIs positioned for each region. Automated shimming was complemented with additional manual shimming (using a full width at half maximum value below 20 Hz for the unsuppressed water signal as a criterion) to optimize spectral resolution. GABA+ and Glx levels were measured using the MEGA‐PRESS (Mescher–Garwood point‐resolved spectroscopy) sequence (Govindaraju et al. 2000; Marjańska et al. 2013; Tremblay et al. 2014). Spectral data were exported and GABA+, Glx, and tCr values were then quantified as ratios from the difference spectra (edit on minus edit off) using LCModel software (v6.3‐1H, copyright Stephen Provencher, Canada) and basis sets provided by Purdue University.

The study used an updated 3T Siemens Difference Basis Set with values for chemical shifts and J‐GABA coupling constants to estimate GABA+ and Glx levels (Kaiser et al. 2008; Kreis and Bolliger 2012; Near et al. 2013). Total creatine (tCr) reference values for GABA+ and Glx were derived from the “edit‐off” spectra of the MEGA‐PRESS measurement. To enhance quantitation accuracy, the LC‐Model's DKNTMN parameter was adjusted to 0.45, optimizing baseline flexibility to minimize measurement error without compromising SNR (Stock et al. 2023). Only spectra with acceptable shim quality (FWHM of 3–7 Hz for the NAA peak) were included in the analyses, with all three VOIs meeting the Cramér–Rao lower bound (CRLB) criterion of less than 15%. See the original published study by Koyun et al. (2024) for further details.

Statistical analyses used an internal metabolite reference signal (Mikkelsen et al. 2017; Mikkelsen et al. 2019), separately referencing both GABA+ and Glx to tCr in the edit‐OFF spectrum. Furthermore, we divided GABA+/tCr by Glx/tCr to obtain a GABA+/Glx ratio. Of note, the resulting ratios used for further analyses are unitless.

### 2.5 Task

**FIGURE 3:**
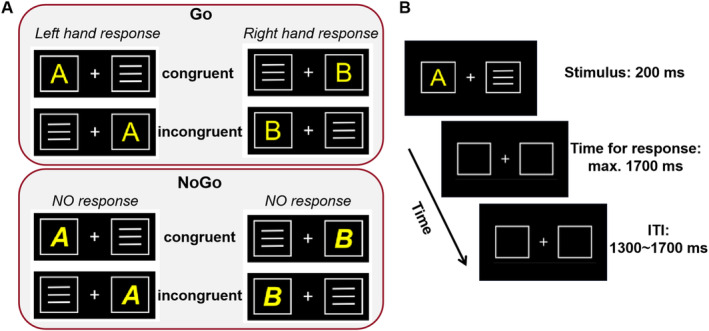
The Simon Go/NoGo task with all possible stimulus configurations. (A) The upper panel displays stimuli in the Go condition. The upper left panel shows stimuli (i.e., “A,” yellow) which require a left‐hand response, while upper right panel shows stimuli (i.e., “B,” yellow) which require a right‐hand response. The lower panel illustrates NoGo stimuli (i.e., “***A***” or “***B***”) that require no response. Trials where the letter stimuli appeared on the same side as their corresponding Go response hand (e.g., “A” on the left, “B” on the right) were labeled as congruent. Trials where the letters appeared on the opposite side (e.g., “A” on the right, “B” on the left) were labeled as incongruent. (B) In each trial, a single letter target stimulus and a contralateral distractor stimulus were simultaneously presented for 200 ms. The maximum time for response was 1700 ms after stimulus presentation and the intertrial interval (ITI) was jittered between 1300 and 1700 ms.

Participants performed a combined Simon and Go/NoGo paradigm (Chmielewski and Beste 2017). Figure 3 illustrates the task design and sequence of events. The task consists of 6 blocks with 120 trials each, totaling 720 trials. These include conditions requiring stronger persistence, like the conflict‐inducing incongruent trials and the less frequent NoGo trials (30%), as well as conditions requiring less persistence, like the nonconflicting congruent trials and more frequent Go trials (70%). Immediately after MPH/placebo administration, participants completed a 16‐trial practice session to familiarize themselves with the task. For the experiment, participants sat around 60 cm from a 24‐in. LCD monitor displaying visual stimuli on a black background. Participants were instructed to respond quickly and accurately to avoid the appearance of the speed‐up prompt (“Faster!”), which occurred in Go trials whenever no response was given within 500 ms after stimulus onset. After each of the six blocks, participants were allowed a self‐timed break before resuming with a button key press. The entire task lasted about 30 min.

### 2.6 EEG Recording and Processing

During the task, EEG signals were captured using 60 equidistant Ag/AgCl electrodes at a sampling rate of 500 Hz with a QuickAmp amplifier (Brain Products GmbH, Gilching, Germany) and BrainVision Recorder software (Version 2.2). All electrodes were referenced to Fpz (*θ* = 90, *φ* = 90), and the ground electrode was placed at *θ* = 58, *φ* = 78. Electrode impedances were kept below 10 kΩ. EEG data preprocessing utilized the *automagic* toolbox (Pedroni et al. 2019) and EEGLAB (Delorme and Makeig 2004) in Matlab 2020a (The MathWorks Corp.). Initially, raw EEG data were downsampled to 256 Hz, and flat channels were removed. Channels were re‐referenced to an average reference, and the PREP pipeline (Bigdely‐Shamlo et al. 2015) was applied to address line noise and discard noisy/bad channels. EOG artifacts were removed using a subtraction method (EOG Regression; Parra et al. 2005). A band‐pass filter ranging from 0.5 to 40 Hz was applied using EEGLAB's pop_eegfiltnew() function, with filter order estimated by default. Additionally, to detect remaining artifactual components, the Multiple Artifact Rejection Algorithm (MARA; Winkler et al. 2011) was applied to automate the independent component analysis (ICA) process. Ultimately, previously discarded channels were interpolated using a spherical method. The preprocessed data was loaded into Matlab 2020a for further analysis using the FieldTrip toolbox (Oostenveld et al. 2011). The EEG data segmentation of Go and NoGo conditions was separately carried out for congruent and incongruent trials. Only correct responses (Go conditions) and omissions (NoGo conditions) were segmented into 4‐s epochs (from −2000 to 2000 ms, locked to the onset of the target letter stimulus). For more details about EEG recording and processing, please see the original published study (Koyun et al. 2024).

### 2.7 Parameterization of the Spectral Data

EEG data were analyzed in two distinct time windows: from 0 to 1000 ms poststimulus presentation (within‐trial period) and from −1000 to 0 ms (pretrial period). Power spectral density (PSD) at each frequency was calculated using Welch's method (0.25 s Hamming window, 50% overlap) (Welch 1967). The calculation was implemented in Matlab using the “pwelch” function. The PSDs were estimated separately for each participant, electrode, condition, and both pretrial and within‐trial periods. For estimating aperiodic activity, the power spectra were parameterized using the Python‐based FOOOF toolbox (version 1.0.0; https://github.com/fooof‐tools/fooof), which decomposes the signal into aperiodic and periodic components (detailed overview of this approach see Donoghue et al. 2020), following the methodology of previous work (Adelhöfer et al. 2021; Gao et al. 2024; Pi et al. 2024; Yan et al. 2024). The FOOOF algorithm conceptualizes the power spectrum as a linear combination of aperiodic activity [*L*(*f*)] and periodic (oscillatory) activity [*G*n(*f*)]. Precisely, the model formula can be written as

$$\mathrm{PSD}\left(f,\;\right)=L\left(f,\;\right)+\sum_{n}G_{n}\left(f,\;\right)$$

where *f* represents the frequency. The PSD is the linear combination of the aperiodic component, *L*(*f*), and n total Gaussians.

The aperiodic component is fit as a function across the entire fitted range of the spectrum. The function for the aperiodic component, *L*(*f*), is defined as

$$L\left(f,\;\right)=b-\log\left[f^{\;x}\right]$$

where *b* represents the aperiodic offset reflecting the broadband power shift, and *x* denotes the aperiodic exponent, equivalent to the slope of the line fitted to the power spectrum in a log–log space.

The periodic (oscillatory) components are characterized as frequency regions of power over and above the aperiodic component. Each oscillatory component, also referred to as “peak” is modeled with a Gaussian profile, defined by three distinct parameters. Each Gaussian fit can be modeled as

$$G_{n}\left(f,\;\right)=a_{n}\exp\left[-\frac{{\left(f-\mu_{n}\right)}^{2}}{2\sigma_{n}^{2}}\right]$$

where *a*_*n*_ is the amplitude, *μ*_*n*_ is the center frequency, and *σ*_*n*_ is the bandwidth of each component.

To obtain a reliable estimation of the aperiodic component, the power spectra data were fitted over a broad frequency range of 3–35 Hz, in accordance with recommendations in the FOOOF documentation. The FOOOF algorithm was configured with the following settings: {aperiodic mode = “fixed,” peak width limits = (2, 8), maximum number of peaks = 8, minimum peak height = 0.05, default settings otherwise}. The power spectra were fit for each electrode, each participant, each task condition, and each period. The *n* = 6 participants whose FOOOF spectra fits (*R* ^2^) were smaller than the group mean minus three times the standard deviation (SD), and aperiodic exponent values that exceeded the group mean ± 3 × SD were also excluded. Additionally, *n* = 4 participants were excluded due to poor MRS data quality. The average *R* ^2^ of spectral fits for all participants (*N* = 91) was above 0.95.

### 2.8 Aperiodic Exponent

The aperiodic parameters encompass both aperiodic exponent and aperiodic offset. The aperiodic exponent was found to be sensitive to index metacontrol states (Zhang et al. 2023). Hence, our analysis primarily focuses on the exponent. Due to the absence of a priori assumptions regarding the scalp distribution of the aperiodic neural activity, we derived the “global” aperiodic‐only signal for each electrode and each participant (Hill et al. 2022). Initially, we averaged the exponent values across 60 electrodes for each participant (Hill et al. 2022) to discern the overall trend of variation. Subsequently, to investigate the distribution of the aperiodic components on the scalp, we conducted an extra cluster‐based permutation test, resulting in statistically significant findings on a global scale. The nonparametric cluster‐based permutation test is a method proposed to localize effects in space, frequency, and time while correcting for multiple comparisons in high‐dimensional EEG/MEG data (Maris and Oostenveld 2007). In this study, clusters were formed based on the adjacency of thresholded sample‐level *F*‐values (*α* = 0.001), with the sum of *F*‐values within a cluster representing the cluster‐level statistics. Significant clusters were determined based on 1000 Monte Carlo random samplings using a significance level of 0.05.

### 2.9 Statistical Analysis

The behavioral data (accuracy and hit response times) as well as the aperiodic exponent and MRS data were analyzed using SPSS software (IBM, version: 27.0). Only trials with correct responses were used for the further analysis of the aperiodic exponent. Behavioral and EEG data was analyzed using repeated‐measures analyses of variances (ANOVAs), where the drug treatment (MPH/placebo), Go/NoGo condition (Go/NoGo), and congruency (congruent/incongruent) were used as within‐subject factors, while MPH dose (low/medium/high) was used as a between‐subject factor. For EEG analyses, time (pretrial/within‐trial period) was used as an additional within‐subject factor.

In order to examine whether baseline GABA+ and/or Glx concentrations predict the catecholaminergic modulation of metacontrol, we ran Pearson's correlation analyses between the already calculated exponent and/or behavioral measures of the congruency effect, the inhibition rates (percentage correct responses), the Go/Nogo effect, and MRS data in different areas (ACC, SMA, striatum).

The *p* values were corrected using the Greenhouse–Geisser and Bonferroni methods whenever appropriate. For all descriptive statistics, the mean and the standard error of the mean (SEM) are reported.

## 3 Results

### 3.1 Behavior

Overall, the common Simon Go/NoGo task effects were replicated: The repeated measures ANOVA for accuracy showed a main effect of Go/NoGo (*F*_(1,98)_ = 135.12, *p <* 0.001, *η*_*p*_^*2*^ = 0.580), with higher accuracies in the Go (95.48% ± 0.308%) than in the NoGo (85.89% ± 0.940%) condition. A main effect of congruency (Simon effect) was also found (*F*_(1,98)_ = 40.04, *p <* 0.001, *η*_*p*_^2^ = 0.290), with higher accuracy in incongruent (91.43% ± 0.566%) than in congruent (89.94% ± 0.589%) trials. Importantly, there was an interaction of Go/NoGo × congruency (*F*_(1,98)_ = 83.38, *p <* 0.001, *η*_*p*_^2^ = 0.460). Post hoc analyses revealed a typical Simon effect, showing higher accuracy in congruent Go (96.25% ± 0.287%) than in incongruent Go (94.71% ± 0.408%) trials. In contrast, in the NoGo condition, higher accuracy in incongruent (88.15% ± 0.925%) than in congruent (83.62% ± 1.010%) trials was found. As for the Go reaction times, a main effect of congruency (*F*_(1,98)_ = 220.24, *p <* 0.001, *η*_*p*_^2^ = 0.692) indicated faster responses in congruent (449.52 ± 3.947 ms) than in incongruent (467.96 ± 4.067 ms) trials.

Regarding drug treatments, the ANOVA for Go reaction times revealed a significant main effect of MPH/placebo (*F*_(1,98)_ = 10.96, *p =* 0.001, *η*_*p*_^2^ = 0.101) indicating that participants responded faster in the MPH condition (454.036 ± 3.911 ms) than in the placebo condition (463.445 ± 4.482 ms), which is consistent with our previous findings (Gao et al. 2024). No interaction effects were found for Go reaction times (all *F* < 1.456, all *p* > 0.238). Accuracy measures showed neither a main effect (*F* = 2.546, *p* = 0.114) nor interaction effects of MPH/placebo (all *F* ≤ 0.697, all *p* ≥ 0.406). ANOVAs for both accuracy and reaction times showed no main effects (all *F* < 0.219, all *p* > 0.804) or interaction effects involving the between‐subject factor of MPH dose group (all *F* ≤ 2.737, all *p* ≥ 0.070).

### 3.2 Aperiodic Exponents (Brain‐Wide)

**FIGURE 4:**
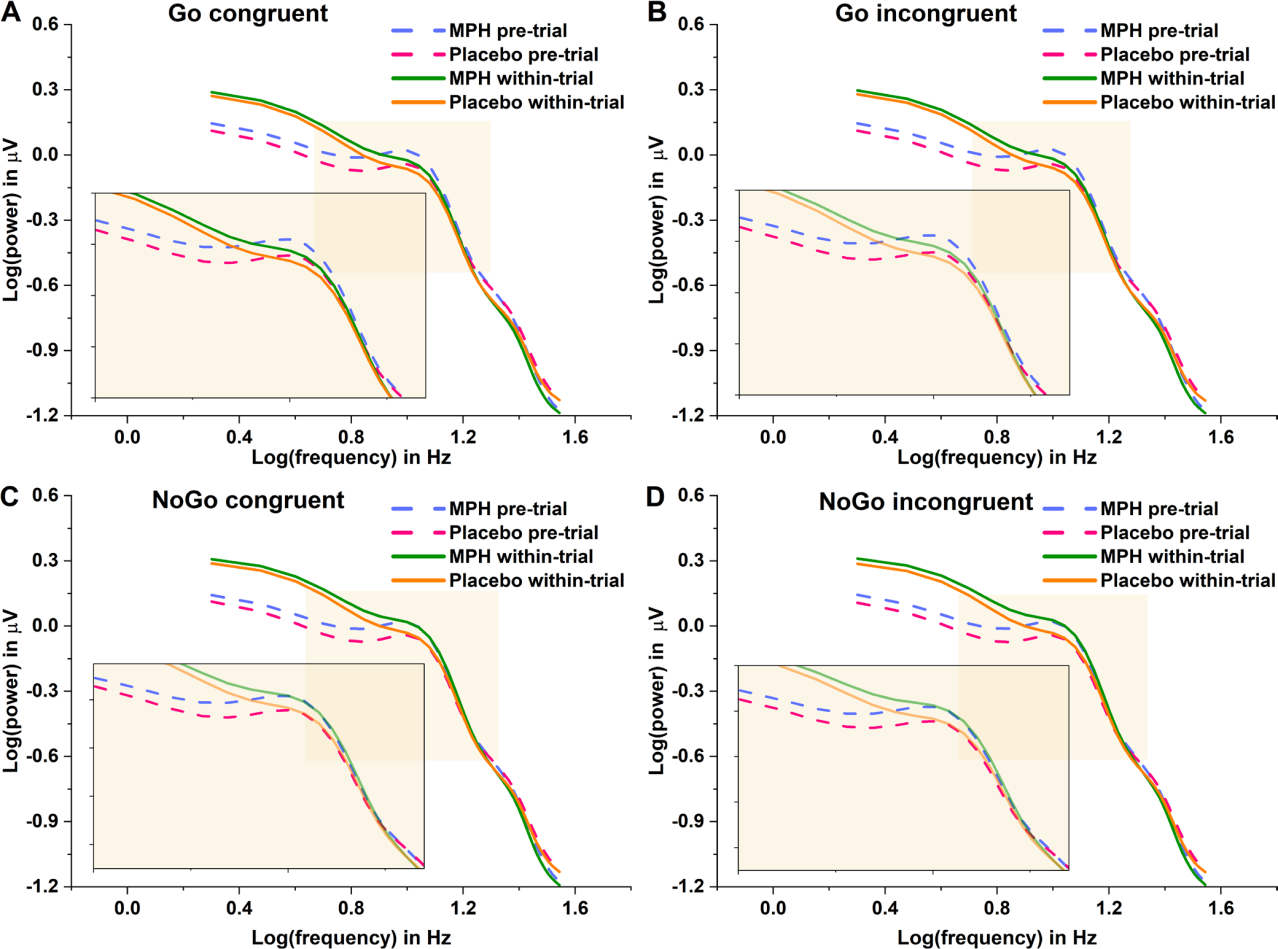
Log–log transformed power spectral density plot after averaging all electrodes and participants. PSDs of MPH and placebo conditions during pretrial and within‐trial for Go congruent (A), Go incongruent (B), NoGo congruent (C) and NoGo incongruent (D) conditions. The beige window in each panel was chosen to better visualize the differences.

The aperiodic component analyses presented below are based on EEG data from *N* = 95 participants, after excluding six outliers identified through FOOOF analyses (see criteria in Section 2). In line with the guidelines of the FOOOF toolbox (Donoghue et al. 2020), PSD within a 3–35 Hz frequency range in log–log space is shown in Figure 4, comparing the MPH and placebo conditions during both within‐trial and pretrial periods across different task conditions. These PSDs were averaged across all electrodes and participants.

After calculating the average aperiodic exponent for all 60 electrodes for each participant, we ran a mixed repeated measures ANOVA. The main effects of time (*F*_(1,92)_ = 1084.69, *p <* 0.001, *η*_*p*_^2^ = 0.922), drug (*F*_(1,92)_ = 27.59, *p <* 0.001, *η*_*p*_^2^ = 0.231), Go/NoGo (*F*_(1,92)_ = 42.07, *p <* 0.001, *η*_*p*_^2^ = 0.314) and congruency (*F*_(1,92)_ = 15.07, *p <* 0.001, *η*_*p*_^2^ = 0.141) were significant, indicating that the exponent was higher (i.e., noise was lower) in the within‐trial than in the pretrial period (1.433 ± 0.019 vs. 1.228 ± 0.019), in the MPH than in the placebo condition (1.367 ± 0.017 vs. 1.294 ± 0.022), in the NoGo than in the Go condition (1.337 ± 0.018 vs. 1.324 ± 0.019), and in the incongruent than in the congruent condition (1.333 ± 0.018 vs. 1.329 ± 0.018). Furthermore, there were significant interactions of drug × time (*F*_(1,92)_ = 6.412; *p =* 0.013; *η* ^2^_*p*_ = 0.065), of Go/NoGo × time (*F*_(1,92)_ = 51.43; *p <* 0.001; *η* ^2^_*p*_ = 0.359), of congruency × time (*F*_(1,92)_ = 10.22; *p =* 0.002; *η* ^2^_*p*_ = 0.100), and of Go/NoGo × congruency × time (*F*_(1,92)_ = 4.398; *p* = 0.039; *η* ^2^_*p*_ = 0.046). All other main effects and interactions were not significant (all *F*_(1,92)_ ≤ 2.598; *p* ≥ 0.080).

**FIGURE 5:**
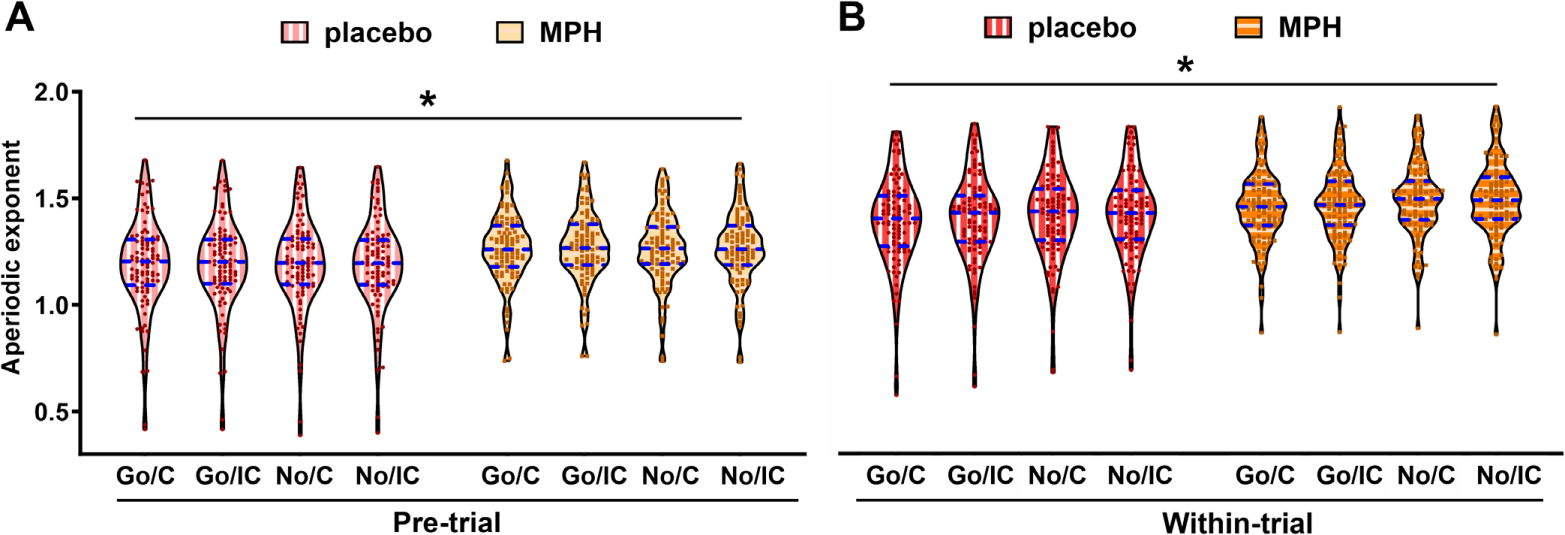
(A, B) The violin plots of the aperiodic exponent under different task conditions: Go congruent (Go/C), Go incongruent (Go/IC), NoGo congruent (No/C), and NoGo incongruent (No/IC). These conditions are analyzed between placebo and MPH administration conditions during pretrial (A) or within‐trial (B) periods. The dots represent individual data points, and the dashed lines indicate the median (middle), upper quartile (top), and lower quartile (bottom). *p < 0.05.

To disentangle this higher‐order interaction involving time, we ran separate ANOVAs for pre‐ and within‐trial periods with the other four factors. For the pretrial, only drug showed a significant main effect (*F*_(1,92)_ = 30.05, *p <* 0.001, *η*_*p*_^2^ = 0.246), with a higher exponent under MPH administration (1.267 ± 0.018) than under placebo administration (1.189 ± 0.022), thus indicating less aperiodic activity (less noise) in the MPH condition than in the placebo condition (Figure 5A). All other main effects and interactions were not significant (all *F* ≤ 2.720; *p* ≥ 0.102). For the within‐trial, the ANOVA uncovered significant main effects of drug (MPH: 1.468 ± 0.018/placebo: 1.399 ± 0.022) (*F*_(1,92)_ = 24.19, *p <* 0.001, *η*_*p*_^2^ = 0.208), of Go/Nogo condition (Go: 1.421 ± 0.019/NoGo: 1.446 ± 0.019) (*F*_(1,92)_ = 52.09, *p <* 0.001, *η*_*p*_^2^ = 0.362), and of congruency (congruent: 1.430 ± 0.018/incongruent 1.437 ± 0.019) (*F*_(1,92)_ = 26.86, *p <* 0.001, *η*_*p*_^2^ = 0.226) (Figure 5B). Moreover, Go/NoGo interacted with congruency (*F*_(1,92)_ = 18.30, *p <* 0.001, *η*_*p*_^2^ = 0.166). No other main effect or interaction was found (all *F* ≤ 2.469; *p* ≥ 0.090).

### 3.3 Aperiodic Exponents (Electrode‐Specific)

In order to identify electrodes that contributed to significant differences across our conditions, we assessed the scalp distribution of the exponent by means of a cluster‐based permutation test.

Specifically, the cluster‐based permutation one‐sample *t* test comparing pretrial and within‐trial of the MPH condition was separately performed for different conditions.

**FIGURE 6:**
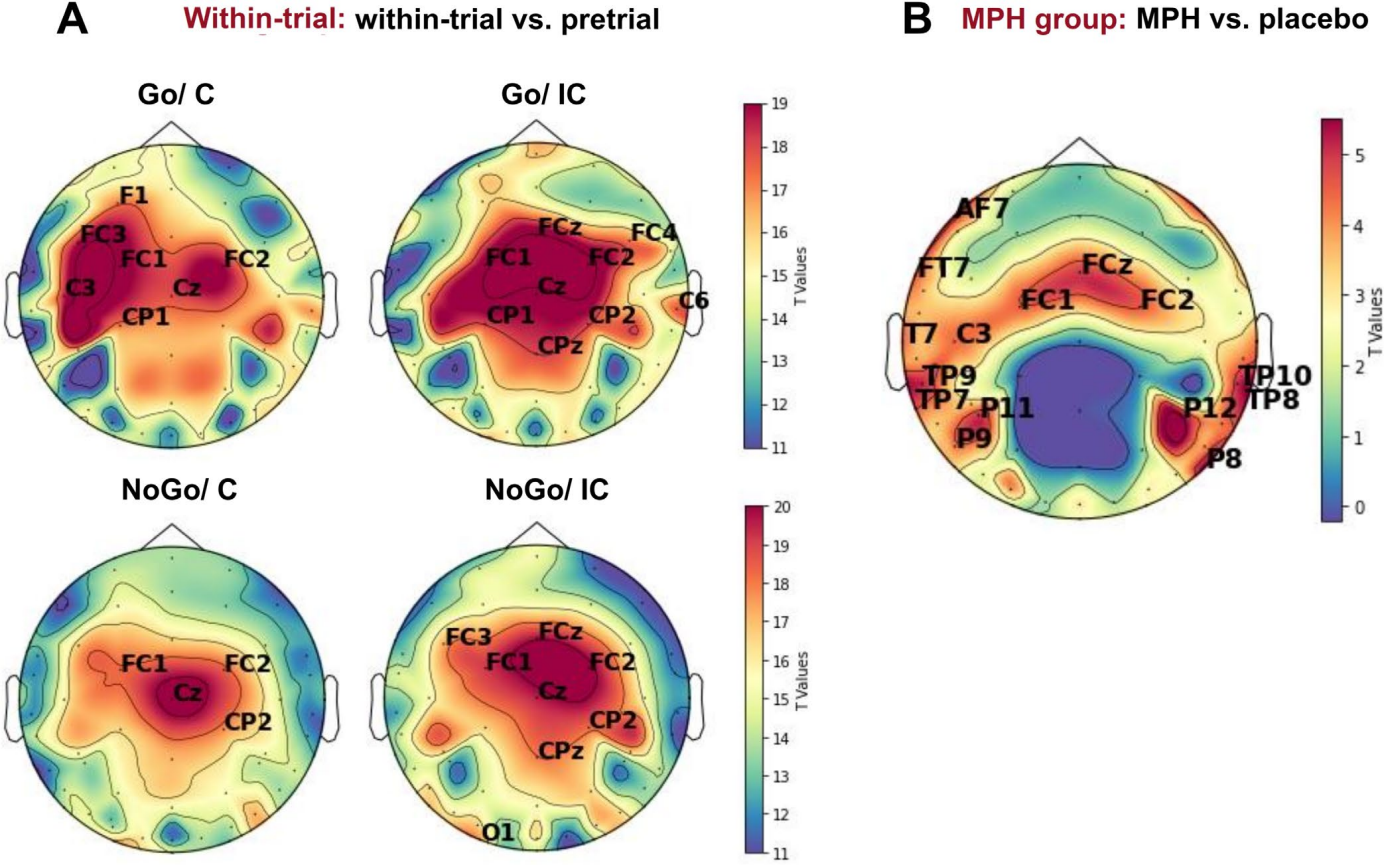
Scalp topographies (A) reveal electrode sites with a significant main effect of time (within‐trial vs. pretrial) across the different task conditions. (B) Electrode sites with a significant drug main effect during the within‐trial period. Labels indicate significant electrode clusters, with colors denoting the sum of cluster‐level *t* values.

Under MPH administration, the pre/within‐trial effect was evident across a broad range of central electrodes, with significant clusters emerging (all *p* = 0.001; *t*_(94)_ ≥ 2.115 in Go congruent, *t*_(94)_ ≥ 3.833 in Go incongruent, *t*_(94)_ ≥ 2.751 in NoGo congruent and *t*_(94)_ ≥ 4.342 in NoGo incongruent) (Figure 6A). As the drug effect was identified in the within‐trial period at the “global” level, the analysis comparing MPH versus placebo was performed during the within‐trial period (all *p* = 0.001; *t*_(94)_ ≥ 2.001) (Figure 6B).

Since electrodes FCz, FC1, and FC2 were concurrently identified in three of the four different conditions in Figure 6A, and also in Figure 6B, this indicates that aperiodic exponents at these sites exhibited the most significant changes. Therefore, focused statistical analysis was warranted for these electrodes.

**FIGURE 7:**
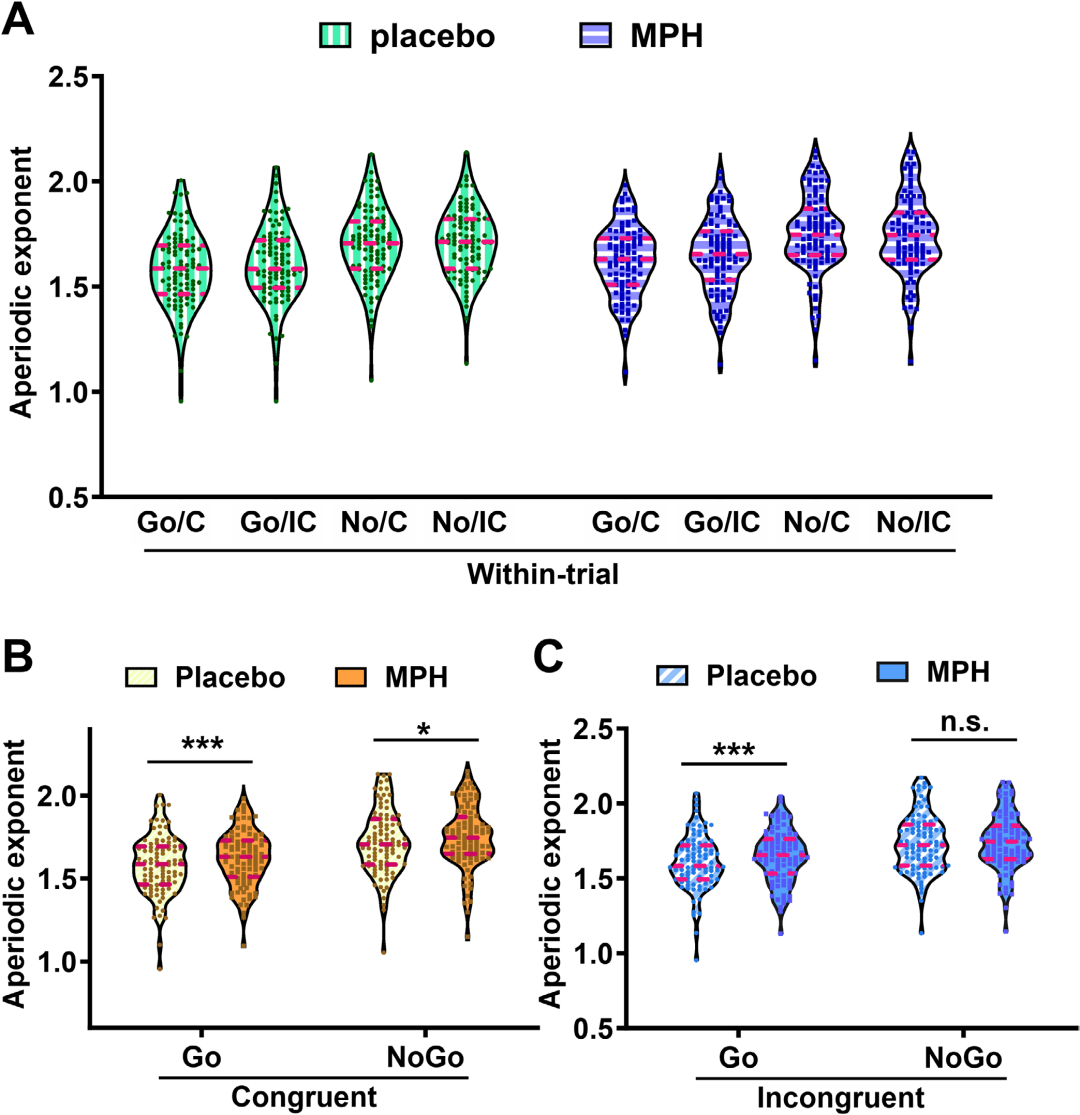
Descriptive results for the aperiodic exponents averaged for electrodes FCz, FC1, and FC2 across Go/congruent (Go/C), Go/incongruent (Go/IC), NoGo/congruent (No/C) and NoGo/incongruent (No/IC) during within‐trial periods. The violin plots represent the distribution of aperiodic exponents, with dots represent individual data points and the dashed lines indicate the median (middle), upper quartile (top), and lower quartile (bottom). *p < 0.05, n.s., no significance.

Based on the averaged aperiodic exponents of electrodes FCz, FC1, and FC2 for each participant and each condition, we performed a repeated measures ANOVA during the within‐trial period. It identified main effects of drug (*F*_(1,92)_ = 8.220, *p =* 0.005, *η*_*p*_^2^ = 0.082), Go/NoGo (*F*_(1,92)_ = 244.56, *p <* 0.001, *η*_*p*_^2^ = 0.727), congruency (*F*_(1,92)_ = 38.04, *p <* 0.001, *η*_*p*_^2^ = 0.293) (Figure 7A). Importantly, there was an interaction of drug × Go/NoGo × congruency (*F*_(1,92)_ = 5.293, *p =* 0.0224, *η*_*p*_^2^ = 0.054). Moreover, three other interactions emerged: first, Go/NoGo × congruency interaction (*F*_(1,92)_ = 22.47, *p <* 0.001, *η*_*p*_^2^ = 0.196); second, drug × Go/NoGo interaction (*F*_(1,92)_ = 12.36, *p <* 0.001, *η*_*p*_^2^ = 0.118); third, drug × congruency interaction (*F*_(1,92)_ = 5.372, *p =* 0.023, *η*_*p*_^2^ = 0.055).

The highest interaction (drug × Go/NoGo × congruency) was further disentangled. That is, in congruent trials, the drug effect (i.e., the difference between MPH and placebo) was significant both in the Go condition (MPH 1.622 ± 0.017 vs. placebo 1.582 ± 0.018, *p <* 0.001) and in the NoGo condition (MPH 1.750 ± 0.019 vs. placebo 1.721 ± 0.021, *p =* 0.016) (Figure 7B). In incongruent trials, the drug effect was significant in the Go condition (MPH 1.648 ± 0.018 vs. Placebo 1.607 ± 0.019, *p <* 0.001), but not in the NoGo condition (MPH 1.743 ± 0.020 vs. Placebo 1.736 ± 0.020, *p =* 0.519) (Figure 7C). No other main effects or interactions were found (all *F*_(1,92)_ ≤ 2.012; *p* ≥ 0.140).

### 3.4 Correlations

**FIGURE 8:**
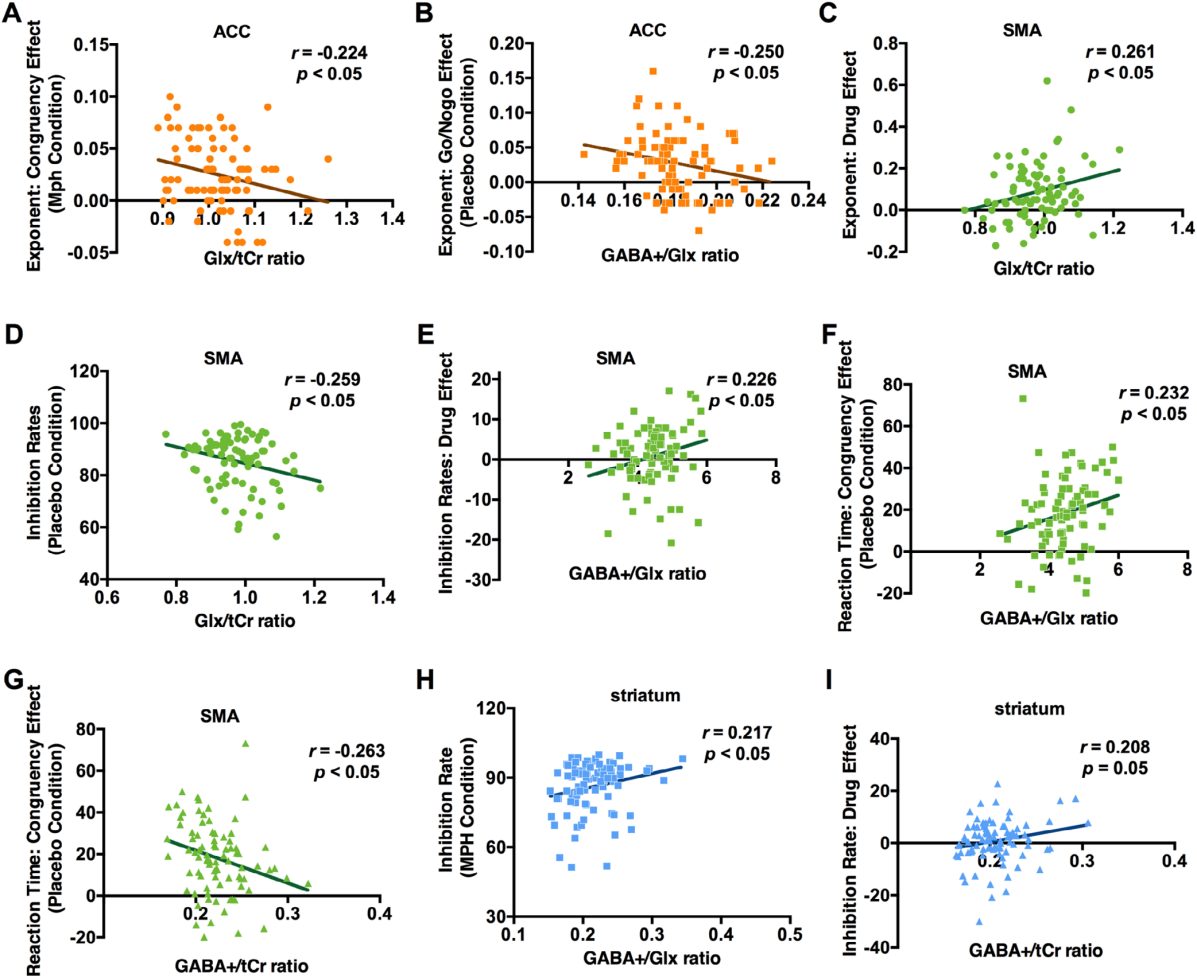
Scatter plots with linear fitted curves for the significant Pearson's correlations between distinct parameters. The displayed *r* values represent Pearson's correlation coefficients. The *p* values correspond to the significance of Pearson's correlation coefficients.

We found several significant correlations of the MRS data with the other assessed data: Two correlations were related to the ACC: a negative correlation between the Glx/tCr ratio in the ACC and the congruency effect of the exponent in the MPH condition, *r*_(87)_ = −0.224, *p* < 0.05 (Figure 8A), and a negative correlation between the GABA+/Glx ratio and the Go/Nogo effect of the exponent in the placebo condition, *r*_(87)_ = −0.250, *p* < 0.05 (Figure 8B).

Five correlations were related to the SMA: a positive correlation between the Glx/tCr ratio and the drug effect on the aperiodic exponent (MPH minus the placebo condition), *r*_(83)_ = 0.261, *p* < 0.05 (Figure 8C), a negative correlation between the Glx/tCr ratio and the inhibition rates in the placebo condition, *r*_(83)_ = −0.259, *p* < 0.05 (Figure 8D), a positive correlation between the GABA+/Glx ratio and the drug effect on inhibition rates (inhibition rate under MPH minus inhibition rate in the placebo condition), *r*_(83)_ = 0.226, *p* < 0.05 (Figure 8E), a positive correlation between the GABA+/Glx ratio and the congruency effect in RT in the placebo condition, *r*_(83)_ = 0.232, *p* < 0.05 (Figure 8F), and a negative correlation between the GABA+/tCr ratio and the congruency effect in RT in the placebo condition, *r*_(84)_ = −0.263, *p* < 0.05 (Figure 8G).

Finally, two correlations were related to the striatum: a positive correlation between the GABA+/Glx ratio and the inhibition rates in the MPH condition, *r*_(83)_ = 0.217, *p* < 0.05 (Figure 8H), and a positive correlation between the GABA+/tCr ratio and the drug effect on inhibition rates (inhibition rate under MPH minus inhibition rate in the placebo condition), *r*_(89)_ = 0.208, *p* = 0.05 (Figure 8I).

## 4 Discussion

The present study had two primary aims. First, we tested whether the previous observations of (Gao et al. 2024) regarding the impact of MPH on the aperiodic exponent in task conditions that call for different kinds of metacontrol could be replicated. Indeed, this could be confirmed. Specifically, MPH increased the aperiodic exponent during both the pretrial period and the within‐trial period, which indicates a state‐like effect. If the aperiodic exponent can be taken to indicate persistence and flexibility biases of metacontrol, this suggests that the drug induces a longer‐lasting persistence bias and thus renders information processing more focused and more selective. Moreover, MPH increased the aperiodic exponent in the within‐trial period and interacted with the two task factors. This under‐additive pattern of this interaction precisely replicates the findings of (Gao et al. 2024) and suggests an upper limit to the impact of MPH: more persistence‐demanding conditions (i.e., Nogo and incongruent conditions) increase the exponent, and this increase adds to the MPH‐induced increase—but at some point, adding another factor does not further increase the exponent. This might point to a physical limitation, in the sense that neural noise can be reduced but only to a certain extent. Taken altogether, these observations are fully consistent with and successfully replicate the findings of (Gao et al. 2024), which implies that the found impact of MPH on the aperiodic exponent is robust and reliable.

The second aim of our study concerns our GABA+ and Glx measures. We were interested in seeing whether, to which degree, and in which sense these measures, and the individual characteristics they imply, can predict behavior and (changes in) metacontrol biases.

Measures related to the ACC were involved in two significant correlations: the GABA+/Glx ratio was negatively correlated with the Go/Nogo effect in the exponent of the placebo condition, and the Glx/tCr ratio was negatively related to the congruency effect in the MPH condition. Given that the direction and sign of the respective correlations in the two other (i.e., MPH and placebo, respectively) conditions were similar, we are reluctant to make much of the condition‐related differences in significance. The two findings suggest that individual differences in baseline amino acid levels in the ACC (Duncan et al. 2011; Duncan et al. 2014; Enzi et al. 2012; Northoff et al. 2007) have an impact on how strongly people respond to persistence‐heavy task challenges, which are consistent with the findings that GABA levels in the ACC, as well as glutamatergic projections from the ACC to the striatum, play an important role in response inhibition (Li et al. 2020; Naaijen et al. 2018; Silveri et al. 2013; Wolff, Chmielewski, et al. 2019). It is interesting to see that the implications of the two significant correlations seem opposite to each other, in the sense that a reduced response to different metacontrol challenges with respect to the Go/Nogo manipulation is driven by (GABAergic) inhibition while a reduced response to different metacontrol challenges with respect to the congruency manipulation is driven by (glutamatergic) excitation. However, GABA and glutamate have opposing effects in terms of neuronal inhibition and excitation, and opposite effects of GABA and glutamate in the ACC have been reported before (see Kiemes et al. 2021), like the observation that impulsivity is negatively correlated with GABA, but positively correlated with glutamate in the ACC (Ende et al. 2016). The fact that the ACC is related to metacontrol is unsurprising. The ACC is assumed to monitor for and pick up signals indicating response conflict, with the aim to initiate processes that increase goal‐related selectivity (Botvinick et al. 2001). From a metacontrol perspective, this amounts to an increase in metacontrol persistence in case of response conflict or stimuli indicating such conflict. GABA and glutamate are likely to be involved in modulating the level of activation of the ACC and/or the noisiness of the signals it receives (Kiemes et al. 2021), which would render a relationship between GABA and glutamate in the ACC on the one hand and metacontrol expressions in the aperiodic exponent on the other very reasonable indeed.

Measures related to the SMA were involved in five significant correlations. First, the positive correlation between Glx/tCr ratios and the drug effect on the aperiodic exponent suggests that individuals with higher SMA Glx levels (stronger excitatory tone) show greater aperiodic changes in response to catecholaminergic stimulation. Hence, the SMA's baseline excitatory tone seems to increase the sensitivity of aperiodic activity to catecholaminergic drugs. Second, the negative correlation between the Glx/tCr ratio and the inhibition rates in the placebo condition suggests that higher excitatory activity in the SMA disrupts the ability to effectively inhibit incorrect responses—which is in line with the ratio's role and the importance of neurochemical states in the SMA (de Joode et al. 2023).

Third, the positive correlation between the GABA+/Glx ratio in the SMA and the drug effect on inhibition rates (inhibition rate under MPH minus inhibition rate in the placebo condition) suggests that strong baseline inhibitory capacities in the SMA create a neural environment where MPH can exert its effects and improve inhibition more effectively (Boy et al. 2010). Fourth and fifth, we found two significant correlations involving the congruency effect in RT in the placebo condition, one positive with the GABA+/Glx ratio and one negative with the GABA+/tCr ratio. The positive correlation may suggest that excessive inhibition can hinder metacontrol “adaptability” to conditions, which may have slowed down conflict resolution. Like the ACC results, this pattern reflects that GABA and glutamate act as opposite forces in terms of neuronal excitability (Ende et al. 2016; Kiemes et al. 2021). In any case, it implies that, under placebo conditions, the congruency effect in RT depends on the participant's baseline neurochemical state.

Finally, measures related to the striatum were involved in two significant correlations. First, the positive correlation between GABA+/Glx ratios and the inhibition rates in the MPH condition indicates that higher baseline GABA+ concentrations or lower Glx concentrations in the striatum may prime neural circuits for efficient modulation under MPH. Second, and relatedly, the positive correlation between GABA+/tCr ratio and the drug effect on inhibition rates (inhibition rate under MPH minus inhibition rate in the placebo condition) supports the role of GABA in the striatum in predicting response inhibition performance (Quetscher et al. 2015; Yildiz et al. 2014).

In summary, our findings underline the idea that aperiodic activity reflects metacontrol processes in general and biases of metacontrol in particular. They also provide evidence for an important role of individual characteristics and dispositions as reflected by GABA+ and Glx baseline levels in the ACC, the SMA, and the striatum. Given that the ACC and the striatum are also suspected to contribute to the regulation of metacontrol, our observations point to an important role of catecholamines in the regulation of metacontrol and task‐specific metacontrol biases.

## Author Contributions

**Yang Gao:** conceptualization, formal analysis, visualization, writing – original draft. **Anna Helin Koyun:** conceptualization, investigation. **Ann‐Kathrin Stock:** conceptualization, investigation, funding acquisition. **Annett Werner:** software, investigation, formal analysis, writing – reviewing and editing. **Veit Roessner:** funding acquisition, writing – reviewing and editing. **Lorenza Colzato:** conceptualization, writing – original draft, writing – reviewing and editing. **Bernhard Hommel:** conceptualization, writing – original draft, supervision, funding acquisition, writing – reviewing and editing. **Christian Beste:** conceptualization, writing – original draft, writing – reviewing and editing, supervision, funding acquisition, writing – reviewing and editing. All authors had full access to the data, gave final approval for publication, and agreed to be held accountable for the work performed therein.

## Ethics Statement

All participants provided written informed consent and received a financial reimbursement for their participation in the study. The ethics committee of TU Dresden approved this study.

## Consent

The authors have nothing to report.

## Conflicts of Interest

The authors declare no conflicts of interest.

## Data Availability

The data that support the findings of this study are available from the corresponding author upon reasonable request.
